# Supplementary figures and images for: The VIL gene CRAWLING ELEPHANT controls maturation and differentiation in tomato via polycomb silencing
Source: PLoS Genet. 2022 Mar 7;18(3):e1009633. doi: 10.1371/journal.pgen.1009633 (PMC8939788; doi:10.1371/journal.pgen.1009633)

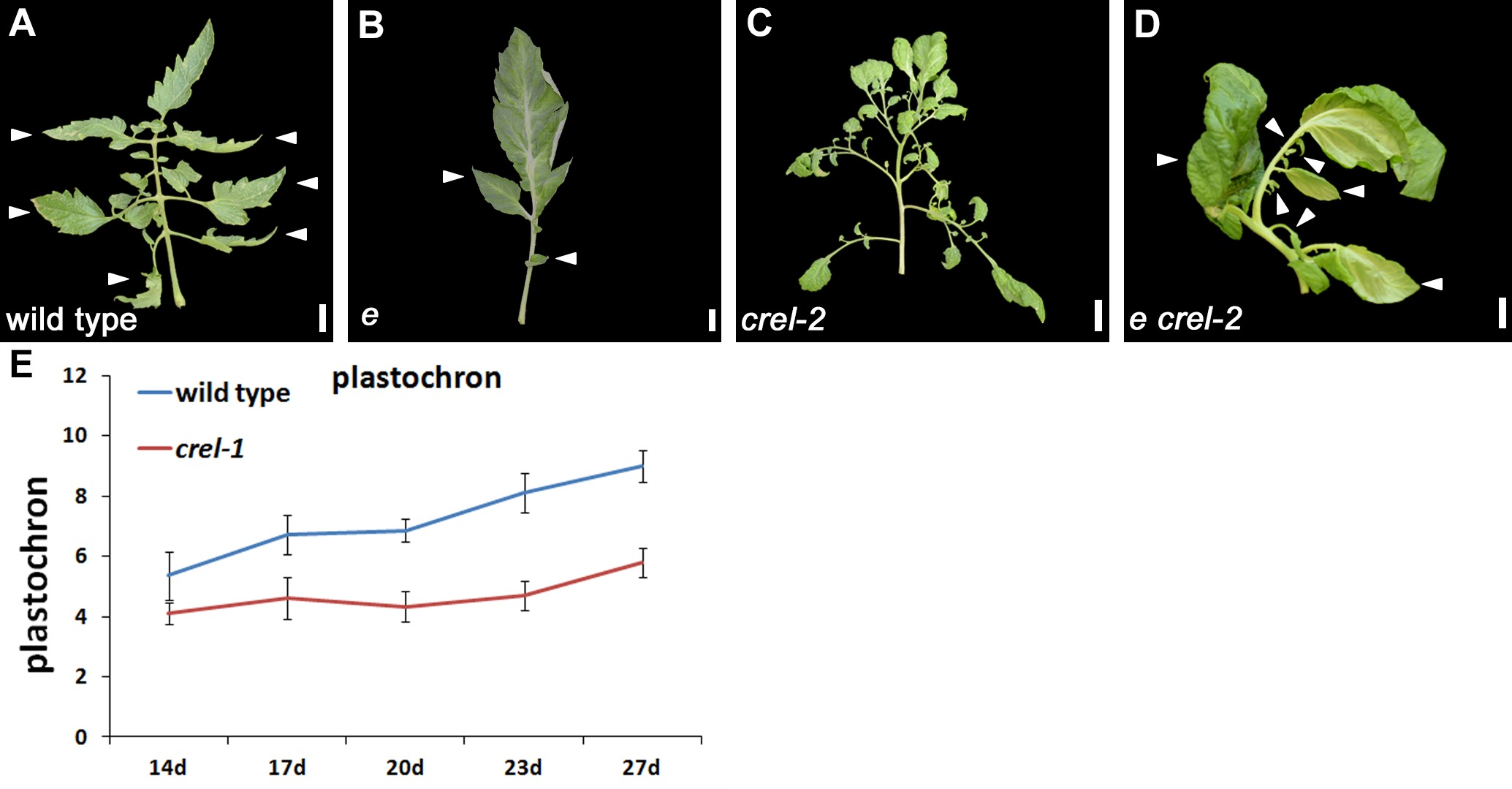

Supplement: S1 Fig — (A-D) Mature 5th leaves of the indicated genotypes.White arrowheads point to primary and intercalary leaflets. Scale bars: 2cm. (E) Slower leaf production in crel-2. The Y axes shows the developmental stage (plastochron, P) of the fifth leaf produced by the plant at the indicated days after seeding. Error bars indicate SD (n = 5–11). (TIF) [file pgen.1009633.s001.tif]

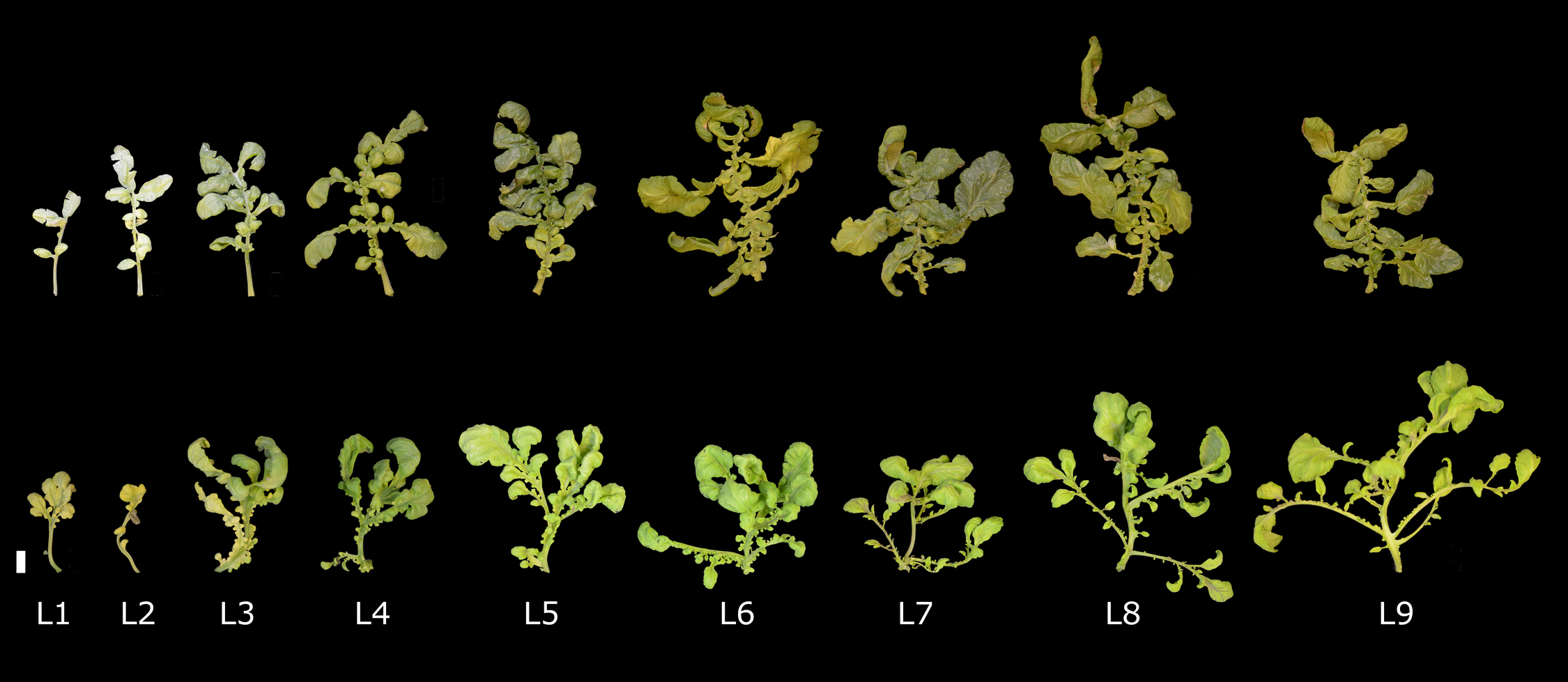

Supplement: S2 Fig — Mature 1st– 9th (L1-L9) of FIL<>miR160 (top) and crel-1 FIL>>miR160 (bottom). Scale bars: 2cm. (TIF) [file pgen.1009633.s002.tif]

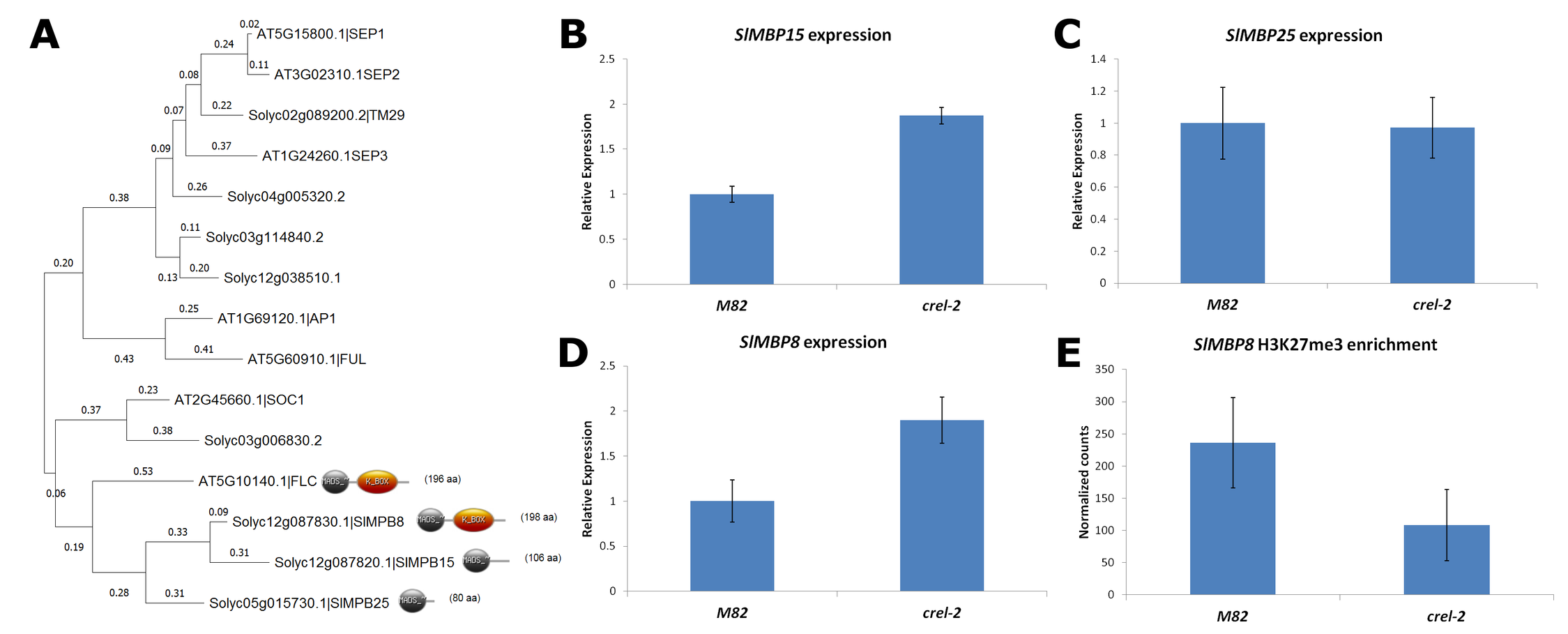

Supplement: S3 Fig — (A) A phylogenetic tree of the Arabidopsis and tomato FLC-MADS BOX proteins, constructed using MEGA X [54,55] using a Maximum Likelihood method. For FLC, SlMBP8, SlMBP15 and SlMBP25 protein lengths and domains are illustrated. (B-D) qRT-PCR analysis comparing the mRNA expression of SlMBP8 (Solyc12g087830), SlMBP15 (Solyc12g087820) and SlMBP25 (Solyc05g015730) in whole shoots containing the SAM and 5 youngest leaf primordia from wild type (WT) and crel-2 plants. SlMBP8 mRNA expression levels were upregulated in crel-2 compared to the WT. Error bars represent the SD of at least three biological replicates. (E) H3K27me3 enrichment -5434 upstream of the SlMBP8 gene in wild type and crel-2 shoot apices. C H3K27me3 was lost in crel-2 in comparison to the wild type. Bars represent the average of 2 biological replicates, and error bars indicate SD. (TIF) [file pgen.1009633.s003.tif]

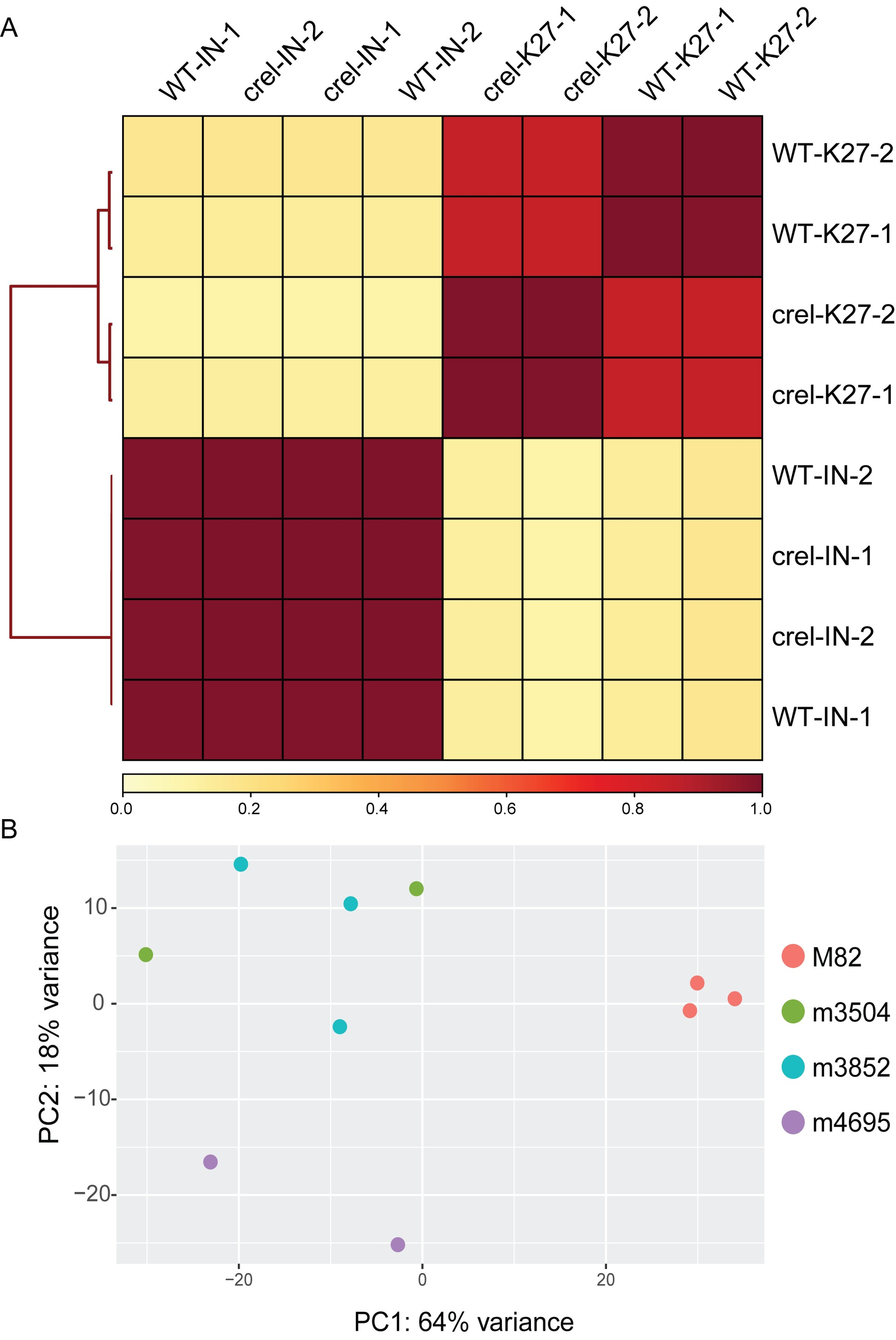

Supplement: S4 Fig — Principal component analysis of input DNA and ChIP-seq samples. (TIF) [file pgen.1009633.s004.tif]

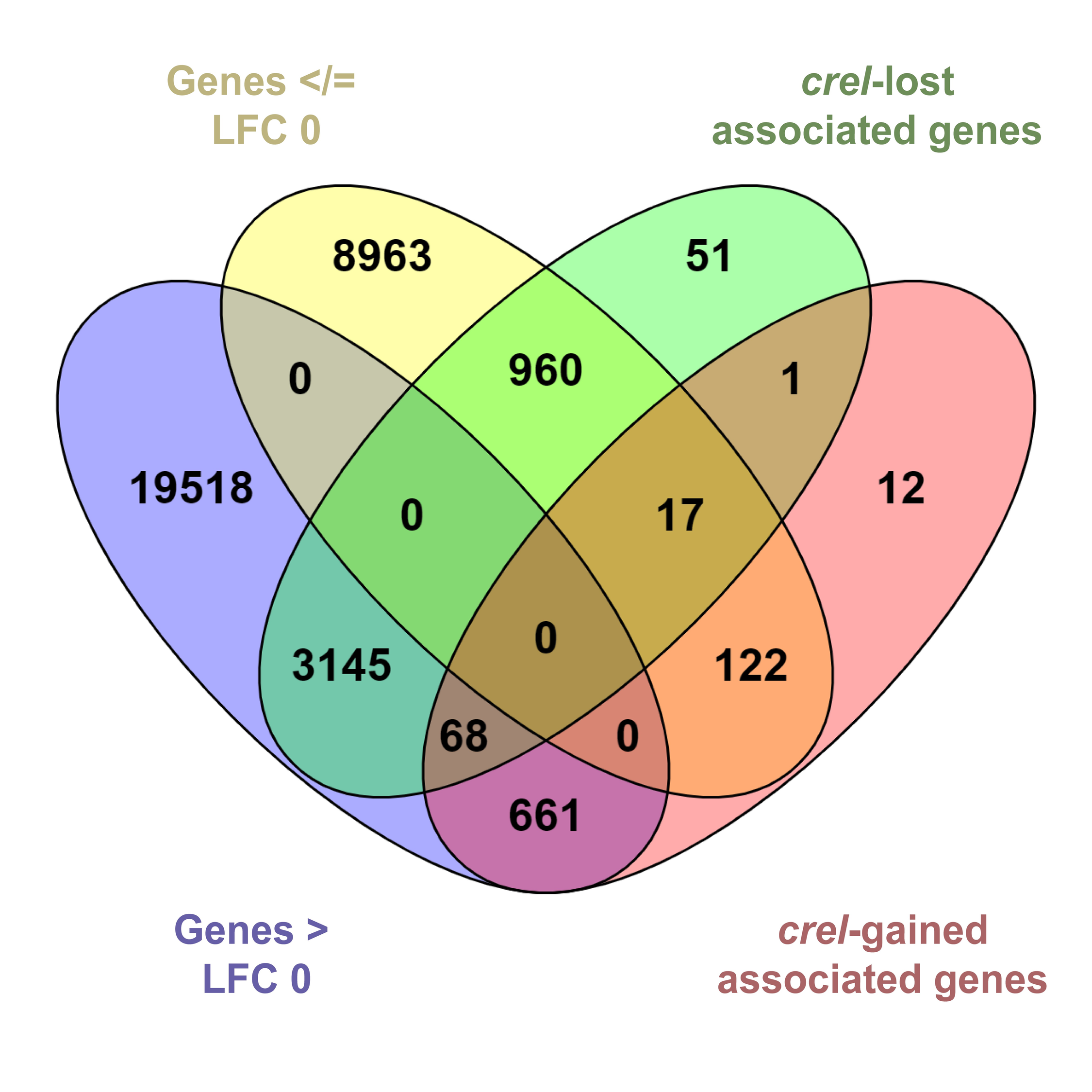

Supplement: S5 Fig — This Venn Diagram shows the overlap between all transcripts detected by RNA-seq with log2 fold change equal to or less than 0 (yellow) and transcripts with log2 fold change over 0 (blue) compared to genes associated with H3K27me3 peaks unique to WT (crel-lost sites; green) and unique to crel-2 (crel-gained sites; red). (TIF) [file pgen.1009633.s005.tif]
