# Supplementary material for: The VIL gene CRAWLING ELEPHANT controls maturation and differentiation in tomato via polycomb silencing
Source: PLoS Genet. 2022 Mar 7;18(3):e1009633. doi: 10.1371/journal.pgen.1009633 (PMC8939788; doi:10.1371/journal.pgen.1009633)
Supplement: S1 Table — (DOCX) [file pgen.1009633.s006.docx]

**Shwartz et al.**

**S1 Table. Primers used in this work**.

| **Primer** | **Sequence 5'->3'** | **Purpose** |
| --- | --- | --- |
| op:VIN3 F | CACCAAGCTTATGTCTGAGCAATTC  CCAAA | Cloning *CREL*  Promoter |
| op:VIN3 R | CTCGAGTTAATGCCAAAGCTTGCAA  CTTT |  |
| p vin3 3000bp-F-3 | CACCTCTAGATAAGTGGGACCCAAT  ATGG | Cloning *CREL*  Promoter |
| p vin3 3000bp R | AAGCTTTGCACTAGGATAAAATCAA  TAGC |  |
| Zach 5 L | CTTTTAGTTTGCAGGCAAGT | Mapping *CREL* |
| Zach 5 R | CTCTAAGGAATGGGATGACA |  |
| Zach 7 L | CACATTGCAAATCGT TTTTA | Mapping *CREL* |
| Zach 7 R | AAACCATACAAGTAAGATACGAAA |  |
| zach 43.2 L | CCAACATTTATACACTTAGGATC | Mapping *CREL* |
| zach 43.2 R | ACCCAACCACTAACAAAAATAC |  |
| chr5 F2 | CCTAGTTTGACTGAGAACAGA | Mapping *CREL* |
| chr5 R2 | GATCCGATCTCCATCATTGAA |  |
| chr5 F3 | GGACTTCATAGTCACGAGAAA | Mapping *CREL* |
| chr5 R3 | GTGGAGTCTTCGAGAACATTA |  |
| chr5 F4 | GGTTGTGAGGTGTCTTATTCT | Mapping *CREL* |
| chr5 R4 | GCCACCATTGCAGTATAGATA |  |
| Jose dcap 58.1 L | AGTCAGGAACTGCTGGATCAAGCATACCAG | Mapping *CREL* |
| Jose dcap 58.1 R | TGTAAGTAAGATCAGCGATGTTGG |  |
| Vin3 real time F | ATTGAGAAGGGTGATGAATGGC | qRT-PCR |
| Vin3 real time R | TCAGAGGATGCAGCAGCAGATACTT |  |
| 16.AGO 5 F | TGTGCCGACTGATAATCCAA | qRT-PCR |
| 16.AGO 5 L | TGCAAACGTATGGAGGAAAA |  |
| RT-GA20ox-3-F | TGATCAGAAAGGCTCATCACAC | qRT-PCR |
| RT-GA20ox-3-R | GGCTCAATCCCAAAAGTTCC |  |
| 02g080220 pectin R | GCAACGGTTGATAACGGACT | qRT-PCR |
| 02g080220 pectin L | AGGACTTCCTTGGTTGAT |  |
| EXP RT F | TGGGTGTGCCTTTCTGAATG | qRT-PCR |
| EXP RT R | GCTAAGAACGCTGGACCTAATG |  |
| MBP8 RT F | AATGCAGAAATCCAGGTCGC | qRT-PCR |
| MBP8 RT R | TCCTCGAGTTGCCTTTCTGT |  |
| MBP15 RT F | ACAGGACTAGCACACCATGT | qRT-PCR |
| MBP15 RT R | TAGGCAAAGCACGACGAGTA |  |
| MBP25 RT F | AGGCAAGCAACTTTCTCCAA | qRT-PCR |
| MBP25 RT R | AGAATTCAAAGAGGCGTCCG |  |
